# Development and psychometric evaluation of an instrument measuring ambulance nurses’ adherence and attitudes to acute chest pain guidelines

**DOI:** 10.1186/s12912-024-02615-3

**Published:** 2024-12-20

**Authors:** Michael Ulrich Hansen, Slobodan Zdravkovic, Ulf Jakobsson, Vedrana Vejzovic, Malin Axelsson

**Affiliations:** 1 Department of Care Science, Faculty of Health and Society, Malmö University, Malmö, Sweden; 2 Center for Primary Health Research, Department of Clinical Sciences, Lund University, Lund, Sweden

**Keywords:** Ambulance nursing, Chest pain, Clinical guidelines, Instrument development

## Abstract

**Background:**

Effective prehospital care for acute chest pain critically relies on ambulance nurses’ adherence to clinical guidelines. However, current adherence is inadequate, with no instruments available to improve the situation. Therefore, this study aimed to develop and psychometrically evaluate an instrument measuring ambulance nurses’ adherence to and attitudes towards acute chest pain guidelines, and to adapt and test the Attitudes Regarding Practice Guidelines instrument for measuring general attitudes towards guidelines.

**Methods:**

An instrument development design was used. A 49-item Adherence Instrument was initially developed for measuring adherence to and attitudes towards acute chest pain guidelines and the 18-item Attitudes Regarding Practice Guidelines instrument was translated into Swedish. Both instruments were validated through cognitive interviews and expert reviews. To ascertain its reliability, a test‒retest was conducted. The construct validity of the Adherence Instrument was assessed via principal component analysis on the basis of a polychoric correlation matrix.

**Results:**

The developed Adherence Instrument was refined to 18 items and showed strong validity and reliability. Similarly, the Attitudes Regarding Practice Guidelines instrument, refined to 12 items, demonstrated strong validity and reliability. Principal component analysis of the Adherence Instrument identified five components: professional evidence-based practice, assessment of symptoms, confidence in skills, clinical autonomy, and guideline clarity and education. These components accounted for 64.5% of the total variance and demonstrated strong reliability, with an ordinal alpha of 0.84 for the entire scale.

**Conclusion:**

The psychometric properties of the Adherence Instrument were satisfactory and will be useful in prehospital emergency care to measure attitudes and adherence towards acute chest pain guidelines.

## Background

To our knowledge, there is a lack of consistent adherence to clinical guidelines among ambulance nurses regarding the treatment of patients with acute chest pain, particularly regarding aspirin administration, with adherence rates ranging from as low as 7.8% to as high as 62% [1–3]. Despite the establishment of clinical guidelines for treating patients with acute chest pain, adherence to these guidelines continues to be a challenge. While previous research has examined factors that may impact adherence to guidelines in general [4], there remains a lack of understanding of the specific reasons why ambulance nurses do not consistently follow the guidelines for treating acute chest pain [5]. Identifying these reasons is crucial for improving patient outcomes and optimizing care.

Various factors may contribute to low adherence to guidelines in prehospital emergency care in general. These may include patient characteristics, such as age, sex, medical history, and severity of illness [1, 2, 6]; the organization structure and practices of the emergency care system [1, 7]; the complexity and clarity of the guidelines themselves [6, 8]; and the strategies used to implement and promote adherence to the guidelines [7, 9], as well as health care professionals’ attitudes towards these guidelines [10]. Although prehospital acute chest pain guidelines are used in specific circumstances, attitudes regarding guidelines in general have been shown to influence responses to specific guidelines for patients with acute chest pain [6]. It is important to carefully consider and address these factors to improve adherence to guidelines in the prehospital setting. Failure of ambulance nurses to adhere to guidelines can lead to diagnostic delays and improper treatment, potentially resulting in poor patient outcomes [11].

To address this issue, it is essential to use reliable and valid instruments to monitor guideline adherence to identify effective strategies for improving adherence [6]. This can help organizations identify areas for improvement and develop strategies for improved adherence [7]. While some instruments exist for evaluating attitudes in general, such as the Attitudes Regarding Practice Guidelines [6] instrument, there is a need for a specific instrument that assesses factors influencing adherence and attitudes to acute chest pain guidelines. By developing and testing such an instrument, ambulance nurses’ adherence and attitudes towards guidelines can be accurately measured, and specific areas where adherence is low can be identified. This can ultimately lead to better patient outcomes and improved quality of care provided by ambulance nurses in the prehospital setting for patients with acute chest pain.

Therefore, this study aimed to develop and psychometrically evaluate an instrument to measure ambulance nurses’ adherence to and attitudes towards guidelines for patients with acute chest pain. An additional aim was to adapt the Attitudes Regarding Practice Guidelines instrument culturally and to test it for prehospital emergency care use.

## Methods

**Fig. 1:**
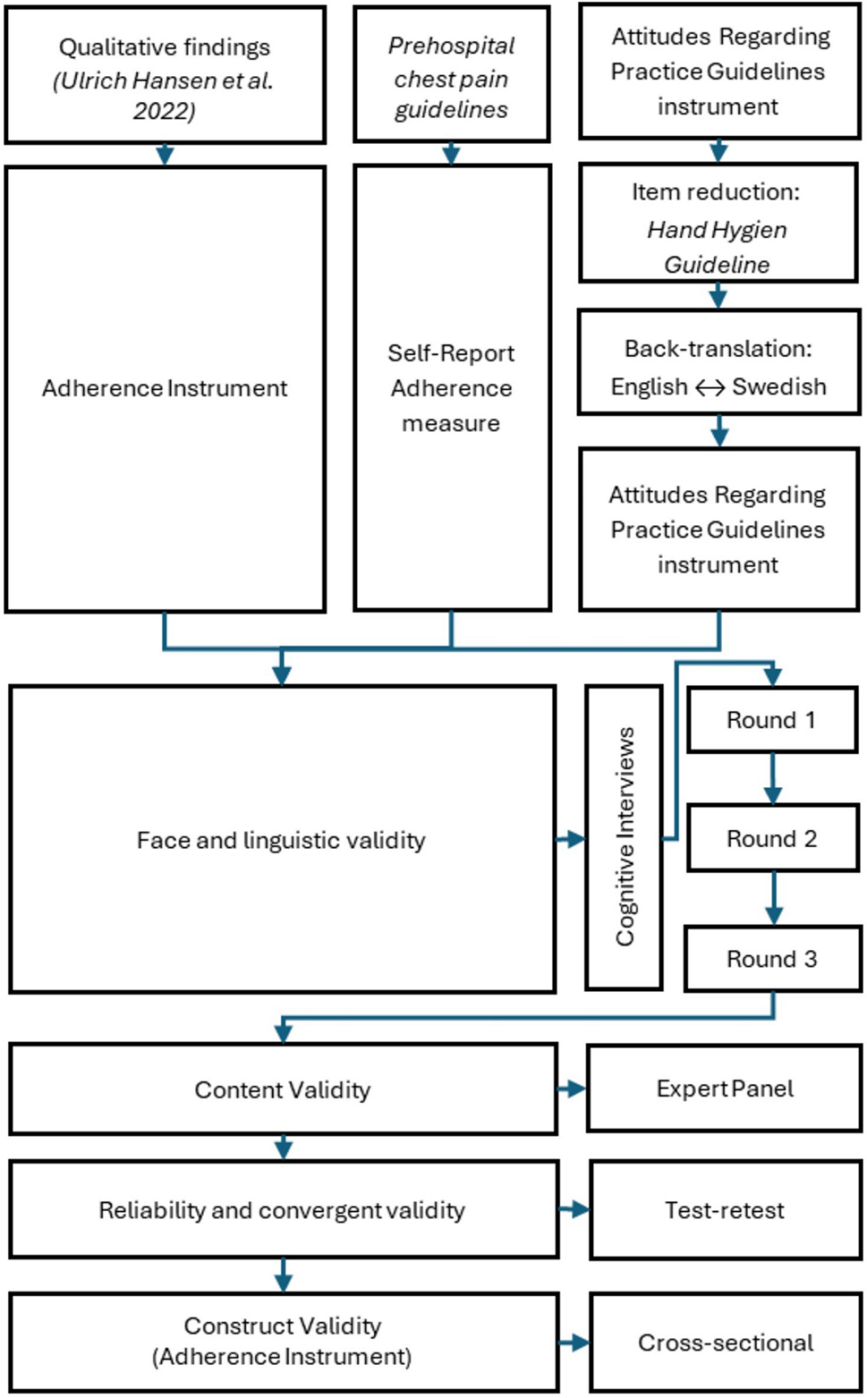
Illustrating the process of instrument development, adaptation, and evaluation for the Adherence Instrument, Self-Reported Adherence measure, and the Attitudes Regarding Practice Guidelines instrument

To develop a reliable and valid instrument for measuring adherence and attitudes among ambulance nurses, a multistep methodological approach was adopted. The conception of the Adherence Instrument was informed by initial interviews conducted with ambulance nurses [5]. On the basis of the insights gathered at this stage, the item pool for the Adherence Instrument was developed. Additionally, an existing instrument, Attitudes Regarding Practice Guidelines [12], was culturally adapted and tested to assess ambulance nurses’ general attitudes towards guidelines. These instruments were validated through cognitive interviews and expert review. Reliability was tested via a test‒retest method. Factor analysis was performed to explore the construct validity of the Adherence Instrument. These steps are described in detail below and are visually represented in Fig. 1.

### Development of the adherence instrument

The Adherence Instrument, developed to assess ambulance nurses’ adherence and attitudes towards acute chest pain guidelines in prehospital emergency care is based on a qualitative study that explored ambulance nurses’ experiences with acute chest pain guidelines [5]. Here, scores range from 18 to 108, where higher scores reflect more positive adherence and attitudes towards guidelines. Initially featuring 49 items rated on a 6-point scale from “strongly disagree” to “strongly agree”, the instrument was refined by the research team for accuracy in reflecting ambulance nurses’ adherence and attitudes to acute chest pain guidelines [13].

### Development of self-reported adherence measure

Self-Reported Adherence measure was assessed using five items rated on a 5-point Likert scale ranging “never” to “always”. Scores range from 5 to 25, with higher scores indicating positive adherence. This section is aligned with expert-developed prehospital protocols for acute chest pain, reflecting established standards for patient care [14].

### Cultural adaptation of the attitudes regarding practice guidelines instrument

The Attitudes Regarding Practice Guidelines instrument [12], initially developed by Cabana et al. [10, 15] and later modified for hand hygiene by Larson [12, 16], is used to identify barriers to guideline adherence among health care professionals by examining their attitudes, beliefs and behaviours. It also aids in validating attitude-related measurements for other instruments. In this study, the instrument was culturally adapted for prehospital emergency care settings through item reduction, back-translation, and testing for validity and reliability. The hand hygiene statements were excluded, and 18 general statements were used for linguistic validation and testing. These statements were back-translated between English and Swedish by bilingual experts to ensure cross-cultural applicability [17]. The final instrument, agreed upon by the research team, consists of 18 items using a 6-point Likert scale ranging from “strongly disagree” to “strongly agree” with score ranging from 18 to 108: higher scores indicate positive attitudes towards guidelines.

### Setting and participants

This study was conducted in the southern part of Sweden, a region with a population of approximately 1.4 million. Participants, including ambulance nurses and physicians, were recruited from all 18 ambulance stations across the region. The ambulance nurses in this study were registered nurses with a three-year university program leading to a Bachelor’s degree, and specialist nurses who additionally obtained a Master of Science (one or two years) in a specialized field. All were actively working in prehospital settings.

### Procedures

To ensure the face, linguistic, and content validity of the instruments, participants were recruited consecutively, and cognitive interviews and expert panel evaluations were conducted. The reliability of the instruments was tested via a test‒retest approach. The construct validity of the Adherence Instrument was assessed through a cross-sectional study design. The study design and methodology are illustrated in Fig. 1 and described below.

#### Cognitive interviews: assessing instruments’ face and linguistic validity

Ambulance nurses were purposively recruited from three ambulance stations for think-aloud cognitive interviews aimed at assessing the facial and linguistic validity of the instruments [18, 19]. Cognitive interviews were conducted in three iterative rounds, with each round including five interviews, as shown in Fig. 1. During the interviews, they were asked to vocalize their thoughts on each item’s clarity and comprehensibility. The researcher noted behaviours such as hesitation or confusion, which were further probed. Two open-ended questions about the instrument’s design were also asked. In total, 15 ambulance nurses participated in these interviews, which were conducted from autumn 2021 to early spring 2022 by the first author MUH. The audio-recorded interviews, ranging 17 to 107 min (median = 72), were transcribed verbatim.

#### Panel of experts: evaluating instruments’ content validity

To evaluate the content validity of the instruments, we adopted the content validity index (CVI) method and assembled a panel of experts [20]. Panel experts, consisting of ambulance nurses and physicians with extensive knowledge of the guidelines, were purposively recruited from three different ambulance stations. Each expert independently appraised the instruments for relevance using a 4-point Likert scale. The expert panel, assembled in the spring of 2022, consisted of seven professionals, including two physicians, four specialist nurses, and one registered nurse.

#### Test-retest: assessing instruments’ reliability

To ascertain the reliability of the instruments, we adopted the test-retest method, with a focus on evaluating their stability and consistency [21, 22]. A participant sample size of 66 participants (accounting for a 25% dropout rate) was determined on the basis of parameters including a hypothesized intraclass correlation coefficient (ICC) of 0.80, α = 0.05, and β = 0.80 [23, 24]. The participants were ambulance nurses who were recruited during their working shifts. The instruments were completed on site, with a second set handed over in a sealed envelope for completion two weeks later. If the second set was not returned within this period, a reminder was dispatched. Recruitment concluded in early autumn 2022, with a total of 67 participants enrolling, 63 completing the initial test, and 46 completing the retest.

#### Cross-sectional study: assessing construct validity

A cross-sectional study was used to assess the Adherence Instrument’s construct validity. The sample size was determined by the recommended 5–10 participants per variable ratio, resulting in a target range of 180 participants for the 18 variables of the instrument [25]. Ambulance nurses from all 18 stations across the region were surveyed. Coordination with the ambulance chief at each station ensured that all ambulance nurses had the opportunity to participate, allowing paper-based surveys to be administered during their shifts and educational events. Data occurred throughout the autumn of 2023. Among the 397 invited participants, 261 participants completed the survey.

### Data analysis

The analysis was performed across four distinct methodological stages of the study. Initially, facial and linguistic validity were assessed through cognitive interviews. Next, content validity was assessed by an expert panel review. A test‒retest approach was used to analyse the reliability of the instruments. The reliability analysis included intrarater, interrater, internal consistency, and convergent validity. Finally, the construct validity of the Adherence Instrument was evaluated using principal component analysis (PCA). The PCA was conducted via the ‘psych package’ in R software 4.4.0 [26], whereas other statistical analyses were carried out using SPSS (version 28) [27].

#### Analysis of instrument validity

Each round of cognitive interviews yielded a descriptive summarization of issues related to item interpretation, phrasing, sequencing, and design, providing essential data for analysing validity [18]. During the analysis, the research group reviewed all transcripts and summaries, focusing on participant-promoted changes in the instruments. Upon analysing and discussing the findings, decisions were made about potential rephrasing, resequencing, redesigning, merging, or deleting of items, or whether to retain them for further evaluation in the next round. A thorough review and analysis of each item ensured systematic data interpretation.

Content validity was measured by the content validity index (CVI), which uses expert panel scores ranging from 1 to 4. Items rated as 3 (quite relevant) or 4 (highly relevant) by experts were considered relevant. The individual item-CVI (I-CVI) and overall scale-CVI (S-CVI) were calculated. The I-CVI is the proportion of experts rating an item 3 or 4. Items were retained if their I-CVI was 0.78 or greater on the basis of seven experts’ input [20, 28]. The S-CVI is the average of the I-CVIs for all items, with a recommended threshold of 0.8 or above [20].

Convergent validity between the constructs of the Adherence Instrument, Self-Reported Adherence measure and Attitudes Regarding Practice Guidelines was assessed via Pearson’s correlation coefficient, which was derived from the test‒retest approach. The strength of the correlation was categorized as follows: a correlation coefficient (r) within the range of 0–0.19 is very weak, 0.20–0.39 is weak, 0.40–0.59 is moderate, 0.60–0.79 is strong, and > 0.80 is very strong [29, 30].

#### Analysis of instrument reliability

The intrarater reliability of the instruments was evaluated through a test‒retest approach using the intraclass correlation coefficient (ICC). The ICC was calculated using a two-way mixed effect model with absolute agreement among the means of k raters. The ICC values are interpreted as follows: 0.5–0.75, moderate reliability; >0.75–0.9, good reliability; and > 0.9, excellent reliability [31]. To evaluate interrater agreement at the item level through test‒retest, Cohen’s linear weighted kappa (K_w_) method was adopted. The interpretations of K_w_ are as follows: 0.00–0.2 slight/poor agreement, 0.21–0.4 fair agreement, 0.41–0.6 moderate agreement, 0.61–0.8 substantial/good agreement, and 0.81–1.0 almost perfect/very good agreement [32, 33]. The internal consistency at the scale level for test-retest reliability was evaluated using Cronbach’s alpha, where values ranging from 0.70 to 0.95 indicate satisfactory to excellent consistency, suggesting that the items withing the scale consistently measure the same underlying construct [34].

#### Analysis of the construct validity of the adherence instrument

To assess the construct validity of the Adherence Instrument, PCA was employed following the structured methodology outlined by Williams et al. [35]. Derived from the cross-sectional study, PCA was initiated by confirming the dataset’s suitability through Barlett’s test of sphericity (*p* < 0.05) and the Kaiser‒Meyer‒Olkin (KMO) measure of sampling adequacy (> 0.5) [35]. Initially, a polychoric correlation matrix was established to handle ordinal data more accurately and reflect the reliability of latent structures [26]. The number of components to retain was initially determined by Kaiser’s criterion (eigenvalue > 1), further supported by the scree test, and validated through parallel analysis [35, 36]. Significant factor loadings were identified from a polychoric correlation matrix via varimax rotation, selecting loadings that exceeded a significant threshold of 0.35 [26, 37]. Internal consistency for the scale and its components identified through the PCA model in our cross-sectional study was determined using the ordinal alpha derived from the polychoric matrix [26]. The correlation strengths were categorized as weak (0.1 to 0.3), moderate (0.4 to 0.6), or strong (0.7 to 0.9) [38].

## Results

The findings are derived from data collected through 15 cognitive interviews, 7 expert panel evaluations, and a test‒retest sequence involving 63 and 46 participants, respectively, focusing on the reliability and validity of the Adherence Instrument, Self-Reported Adherence measure, and Attitude Regarding Practice Guidelines. The construct validity of the Adherence Instrument was assessed using data from 261 participants in a cross-sectional study, as detailed in Table 1.

**Table 1:** Characteristics of the participants in the cognitive interviews, expert panel, test-retest, and cross-sectional studies

	Cognitive interviews	Expert panel	Test-retest		Cross-sectional
**Participants**			**Test**	**retest**	
Number of participants	15	7	63	46	261
**Age in years**					
Range (median)	33–65 (42)	30–58 (35)	24–55 (38)	24–55 (37.5)	25–67 (39.0)
**Sex**					
Men	7	5	43	30	120
Women	8	2	20	16	141
**Education**					
Registered nurses^a^	0	1	14	8	59
Specialist ambulance care nurses^a^	9	3	32	27	148
Specialist anaesthetic care nurses^a^	4	1	11	9	34
Specialist intensive care nurses^a^	0	0	4	1	23
Specialist emergency care nurses^a^	1	0	1	0	5
Other specialist nurses^b^	1	0	1	1	8
Physician - Emergency Medicine^c^	N/A	1	N/A	N/A	N/A
Physician - Cardiologist^c^	N/A	1	N/A	N/A	N/A
**Years of working experience**	Range (median)	Range (median)	Number n (percent %)	Number n (percent %)	Number n (percent %)
Years working in ambulance care	2–22 (9)	1–17 (4)	N/A	N/A	N/A
< 1 year working in ambulance care	N/A	N/A	9 (14.3)	3 (6.5)	18 (6.9)
1–3 years working in ambulance care	N/A	N/A	9 (14.3)	9 (19.6)	42 (16.1)
4–6 years working in ambulance care	N/A	N/A	13 (20.6)	8 (17.4)	31 (11.9)
7–9 years working in ambulance care	N/A	N/A	10 (15.9)	7 (15.2)	33 (12.6)
> 10 years working in ambulance	N/A	N/A	22 (34.9)	19 (41.3)	78 (29.9)

^a^Registered nurse with 3 years of university studies (Bachelor’s programme in nursing) followed by specialist nurses with an additional 1 year or more of university studies;

^b^Other specialist nurses including midwives, psychiatric nurses, surgical nurses, and theatre nurses

^c^Physicians with 6 years of university studies (Medical Programme) followed by at least 5 years residency

*Note* N/A = Not applicable

### Validity of instruments

#### Face and linguistic validity of instruments

A total of 15 ambulance nurses, 53% of whom were women, participated in cognitive interviews to assess the facial and linguistic validity of the instruments. The average age of the participants was 43.6 years, with a standard deviation of 10.5 years. Additionally, participants had, on average, 5.3 years of experience in prehospital emergency care, as detailed in Table 1.

During the three iterative rounds of cognitive interviews, 56 issues were identified that needed further examination to determine whether they were problematic. These issues were grouped into four categories: retained, rephrased, merged, or removed. The initial version of the Adherence Instrument consisted of 49 items. After three rounds of analysis, 37 issues were identified. As a result, four items were retained, 19 items were rephrased, five items were combined, and nine items were removed. For the Self-Reported Adherence measure, the wording of 2 items was revised following three rounds of cognitive interviews. The initial version of the Attitudes Regarding Practice Guidelines instrument consisted of 18 items. After three rounds of analysis, 17 issues were identified and rephrased. Overall, design changes were made to improve comprehension, including increasing the text size, adding borders and lines, clarifying information boxes, and organizing items into categories. For a detailed list of changes made during the cognitive rounds, see Table 2.

**Table 2:** Process of cognitive interview round analysis and findings

	Round 1	Round 2	Round 3
Adherence Instrument and Self-Reported Adherence measure	Item retained: 17 Item rephrased: 23, 30, 31–32, 45, 51–52, 54 Item merged: 31–32, 38–40	Item removed: 16, 23, 39, 40, 47 Item rephrased: 12–13, 21, 25, 27–29, 31, 46, 48 Item retained: 32, 43, 45	Item removed: 45, 47, 54, 60 Item rephrased: 52
Attitudes Regarding Practice Guidelines instrument	Item rephrased: 56, 58, 61–66, 68–70, 72–73	Item rephrased: 54, 58, 60,	Item rephrased: 31
Demographics	Item removed: 10	Item 9 rephrased	-
Overall design changes	Increased text size of information boxes	Rephrasing of information box text for clarification	-
	Increased lining space in between items	Sorting of items into categories for clarity: knowledge, resources, profession, evidence, work environment, self-awareness, design, symptoms, treatment	-
	Remove borders in between items	Added underlining borders in between items	-

#### Content validity of the instruments

The evaluation of the content validity of the Adherence Instrument, Self-Reported Adherence measure, and Attitudes Regarding Practice Guidelines instrument was carried out by a panel of seven content experts, including two physicians, four specialist nurses, and one registered nurse (see Table 1). The average age was 40.0 years, with a standard deviation of 9.3 years and an average of 6.6 years of experience in prehospital emergency care.

The Adherence Instrument initially included 31 items. Following content validity testing, 13 items were removed, as they did not meet the predetermined criteria, leaving 18 items. The Adherence Instrument demonstrated an acceptable S-CVI of 0.920, with the I-CVI ranging from 0.714 to 1.0, as presented in Table 3. However, the Self-Reported Adherence measure had a lower S-CVI of 0.742 than the set criterion of 0.8. A single item with a lower I-CVI of 0.571, which did not meet the set criterion of ≥ 0.78, was nonetheless retained for measurement purposes. The Attitudes Regarding Practice Guidelines, initially consisting of 18 items, had 6 items removed after content validity testing because they did not meet the set criterion of ≥ 0.78. This resulted in 12 items being retained. The instrument displayed an acceptable S-CVI of 0.916, with I-CVI values ranging from 0.857 to 1.0.

**Table 3:** Items were rated 3 or 4 on a 4-point relevance scale for the Adherence Instrument, Self-Reported Adherence measure, and the Attitudes Regarding Practice Guidelines instrument

Item	Exp. 1	Exp. 2	Exp. 3	Exp. 4	Exp. 5	Exp. 6	Exp. 7	No. in agreement	I-CVI	S-CVI
Adherence Instrument										0.920
1	3	4	2	4	4	3	4	6	0,857	
2	3	3	3	2	3	4	4	6	0,857	
3	4	3	4	3	4	3	4	7	1	
4	4	3	4	3	3	4	4	7	1	
5	4	4	3	4	4	4	4	7	1	
6	4	4	4	3	2	3	4	6	0,857	
7	2	4	3	4	3	4	3	6	0,857	
8	4	4	4	2	4	3	4	6	0,857	
9	4	4	4	1	4	3	4	6	0,857	
10	4	4	3	3	4	4	3	6	0,857	
11	4	4	4	4	4	4	4	7	1	
12	3	4	3	3	4	4	4	7	1	
13	3	4	3	2	1	4	4	5	0,714	
14	4	4	3	3	4	3	4	7	1	
15	4	4	3	1	4	4	4	6	0,857	
16	4	4	3	4	4	4	4	7	1	
17	3	4	3	3	3	4	4	7	1	
18	3	4	4	3	4	4	4	7	1	
**Self-Reported Adherence measure**										**0.742**
1	3	2	2	3	4	3	4	5	0,714	
2	3	4	3	3	2	2	3	5	0,714	
3	3	4	3	2	2	2	3	4	0,571	
4	4	4	4	3	2	4	4	6	0,857	
5	4	4	3	3	2	4	4	6	0,857	
**Attitudes Regarding Practice Guidelines instrument**										**0.916**
1	3	2	2	3	4	3	4	5	0,857	
2	3	4	3	3	2	2	3	5	0,857	
3	3	4	3	2	2	2	3	4	1	
4	4	4	4	3	2	4	4	6	0,857	
5	4	4	3	3	2	4	4	6	0,857	
6	4	4	4	3	2	3	4	6	0,857	
7	2	4	3	4	3	4	3	6	0,857	
8	4	4	4	2	4	3	4	6	0,857	
9	4	4	4	1	4	3	4	6	0,857	
10	4	4	3	3	4	4	3	6	0,857	
11	4	4	4	4	4	4	4	7	1	
12	3	4	3	3	4	4	4	7	1	

Footnote Exp = Expert; I-CVI = item content validity index; S-CVI = scale content validity index

#### Convergent validity of the instruments

Convergent validity assessment of the instruments indicated a positive correlation between the Adherence instrument and the Attitudes Regarding Practice Guidelines instrument, with Pearson’s *r* = 0.649 (*p* < 0.001). A positive correlation was also observed between the Adherence Instrument and the Self-Reported Adherence measure, with Pearson’s *r* = 0.309 (*p* = 0.015). However, there was no significant correlation between the Self-Reported Adherence measure and Attitudes Regarding Practice Guidelines instrument (Pearson’s *r* = 0.116, *p* = 0.368).

### Reliability of instruments

The test‒retest interval was, on average, 13.5 days (SD = 1.9 days). Among the 67 participants enrolled in the study, 63 (94.0%) completed the initial test, whereas 46 (67.2%) completed the retest. Among all of the respondents, 64.2% were men, with a mean age of 37.9 years. On average, they had 3.43 years of experience in prehospital emergency care, as shown in Table 1.

The Adherence Instrument had a mean test score of 79.9 (95% CI: 77.8–82.0) out of a possible 108, with an acceptable Cronbach’s alpha of 0.800 and a good reliability, with an ICC = 0.794 (95% CI: 0.628–0.887, *p* < 0.001). Agreement between the test and retest results ranged at the item level from 0.234 to 0.587 k_w_, indicating fair to moderate quality (Table 4). For the Self-Reported Adherence measure, the mean test score was 18.3 (95% CI: 17.7–19.0) of a possible 25, with an acceptable Cronbach’s alpha of 0.746 and a moderate ICC of 0.738 (95% CI: 0.528–0.856, *p* < 0.001). Test‒retest agreement at the item level ranged from 0.330 to 0.508 k_w_, indicating fair to moderate quality (Table 4). The Attitudes Regarding Practice Guidelines instrument had a mean test score of 57.6 (95% CI: 56.0-59.2) out of a possible 72, with an acceptable Cronbach’s alpha of 0.817 and a good reliability, with an ICC = 0.819 (95% CI: 0.673-0.900, *p* < 0.001). Test‒retest agreement at the item level ranged from 0.276 to 0.645 k_w_-value, indicating fair to substantial/good quality (Table 4).

**Table 4:** Cohen’s Linear Weighted Kappa Assessments of the Adherence Instrument, Self-Reported Adherence measure, and the Attitudes Regarding Practice Guidelines instrument

Adherence Instrument				
The following statements relate to your attitudes and experience regarding the application of guidelines in the treatment of patients with acute chest pain during prehospital emergency care.		Cohen’s Kappa (Kw) 95% CI	Standard Error (SE)	P value
1	I find it difficult to keep track of what’s new when the guidelines are updated	0.587	0.082	< 0.001
2.	I receive sufficient education regarding the guidelines	0.483	0.091	< 0.001
3.	I believe it is important that everyone in ambulance care follows the guidelines	0.460	0.116	< 0.001
4.	I feel a professional obligation to follow the guidelines	0.541	0.096	< 0.001
5.	The guidelines help me understand what is expected of me	0.234	0.118	0.025
6.	I believe the guidelines are based on scientific evidence	0.384	0.104	< 0.001
7.	I agree with the content of the guidelines	0.332	0.120	< 0.001
8.	I prefer to rely on my clinical experience rather than the guidelines	0.450	0.092	< 0.001
9.	I prefer to follow the advice of colleagues rather than use the guidelines	0.280	0.122	0.004
10.	I consider myself to have sufficient practical knowledge to use the guidelines	0.281	0.141	0.005
11	I find the guidelines to be unclear	0.364	0.105	< 0.001
12.	I find it difficult to assess whether the patient’s pain is of cardiac origin	0.409	0.100	< 0.001
13.	I become uncertain about the use of the guidelines when encountering patients with nonspecific chest pain	0.263	0.113	0.010
14.	I become uncertain about the use of the guidelines when encountering patients with acute chest pain and several other symptoms.	0.256	0.103	0.006
15.	I believe that only an ECG can determine whether the guidelines should be followed or not	0.551	0.104	< 0.001
16.	The guidelines help me in deciding how to treat patients with acute chest pain	0.271	0.134	0.004
17.	I feel confident in treating patients with acute chest pain according to the guidelines	0.297	0.104	< 0.001
18.	I refrain from following the guidelines if I assess that the treatment may worsen the patient’s condition	0.344	0.092	0.001
**Self-Reported Adherence measure**				
1.	How often do you adhere to the guidelines when caring for patients with acute chest pain in ambulance care?	0.382	0.153	< 0.001
To what extent do you use the following medications when treating patients suspected of acute heart disease in prehospital emergency care?				
2.	Morphine	0.508	0.090	< 0.001
3.	Oxygen	0.502	0.114	< 0.001
4.	Nitro-glycerine	0.330	0.132	0.001
5.	Aspirin	0.458	0.110	< 0.001
**Attitudes Regarding Practice Guidelines instrument**				
The following statements concern your general experience with guidelines.				
1	I’m not familiar with the guidelines at my workplace	0.276	0.098	0.005
2.	There are so many guidelines that it’s almost impossible to keep track of them all	0.633	0.089	< 0.001
3.	At my workplace, the guidelines are easily accessible	0.293	0.108	0.001
4.	I don’t have the time to stay informed about current guidelines	0.430	0.100	< 0.001
5.	The guidelines are practical to use	0.645	0.082	< 0.001
6.	In our organization, guidelines are important	0.375	0.110	< 0.001
7.	In general, the advantages of the guidelines outweigh their disadvantages	0.281	0.116	0.002
8.	The guidelines hinder my professional autonomy	0.383	0.087	< 0.001
9.	I am expected to use guidelines in my work	0.576	0.113	< 0.001
10.	Highlighting guidelines reduces the risk of malpractice	0.322	0.115	0.004
11	Guidelines help to standardize care and ensure that patients are treated in a consistent manner	0.366	0.107	0.001
12.	At my workplace, there is sufficient support and resources to enable the use of guidelines	0.391	0.107	< 0.001

Footnote **Cohen’s linear weighted kappa (Kw)**: Measures agreement; ranges from − 1 (no agreement) to + 1 (perfect agreement). Higher values indicate stronger agreement among the raters. **95% confidence interval (95% CI)**: Range in which the true Kappa value is likely to fall, with 95% certainty. **Standard error (SE)**: Indicates the precision of the kappa estimate. Smaller values indicate more precise estimates. **P value**: Statistical significant. Values < 0.05 indicate significant results

### Construct validity of the adherence instrument

The cross-sectional study achieved a participation rate of 65.7%, with 261 out of 397 distributed questionnaires successfully returned. Within this respondent group, a small portion (*n* = 7) did not complete every item of the Adherence Instrument. Demographic analysis revealed an average participant age of 40.88 years (SD = 9.30), with a distribution of 45.9% male and 54.0% female respondents. Most participants were employed full-time (97.3%), with 77.4% being specialist nurses and 22.6% being general nurses. The participants demonstrated a broad range of experience, predominantly exceeding 10 years in both nursing (56.5%) and prehospital emergency care (over 35%), as outlined in Table 1.

PCA was conducted to identify the underlying components within our dataset. The PCA model identified a five-component solution, as determined by a combination of a scree test, eigenvalues greater than 1, and parallel analysis. These components together accounted for 64.5% of the total variance in the data, as detailed in Table 5. Kaiser‒Meyer‒Olkin’s test returned a value of 0.748, confirming sampling adequacy, whereas Bartlett’s test of sphericity demonstrated the data’s suitability for this analysis with a significance of *p* < 0.001.

**Table 5:** Components extraction via parallel analysis

Component number	Total (actual mean eigenvalue)	Parallel analysis (random mean eigenvalue)	Decision	Variance (%)	Cumulative percent after factor extraction (%)
1	5.102	1.488	Retained	28.347%	28.347%
2	2.606	1.388	Retained	14.478%	42.825%
3	1.383	1.316	Retained	7.687%	50.513%
4	1.304	1.255	Retained	7.246%	57.760%
5	1.223	1.197	Retained	6.796%	64.556%
6	0.957	1.145	Discarded	5.317%	69.874%
7	0.920	1.096	Discarded	5.116%	74.991%
8	0.768	1.046	Discarded	4.272%	79.263%
9	0.726	1.000	Discarded	4.038%	83.301%
10	0.576	0.952	Discarded	3.204%	86.506%
11	0.562	0.911	Discarded	3.125%	89.632%
12	0.475	0.875	Discarded	2.644%	92.276%
13	0.370	0.830	Discarded	2.059%	94.335%
14	0.339	0.790	Discarded	1.885%	96.221%
15	0.273	0.748	Discarded	1.517%	97.738%
16	0.215	0.702	Discarded	1.198%	98.937%
17	0.111	0.656	Discarded	0.617%	99.554%
18	0.080	0.595	Discarded	0.445%	100.000%

Footnote Table 5 illustrates the actual mean eigenvalue against random mean eigenvalues from parallel analysis, assisting in the decision to retain or discard components. The components are retained if their actual mean eigenvalue exceeds the random mean eigenvalue

Following component extraction, a varimax rotation was applied to refine the clarity and distinctiveness of the item loadings. The adjusted loadings, presented in Table 6, facilitated the identification and labelling of five distinct rotated components (RC1 to RC5). These rotated components were designated as follows: professional evidence-based practice (RC1), assessment of symptoms (RC2), confidence in skills (RC3), clinical autonomy (RC4), and guideline clarity and education (RC5). The labelling of these components was achieved through a consensus among the research team based on iterative discussions and reflection that considered both the statistical significance and thematic relevance of the factor loadings. This method follows the guidance of factor labelling as a subjective and inductive process, as discussed by Henson and Roberts [39] and supported by Williams et al. [35], ensuring that the labels accurately represent the underlying constructs of the data analysed.

**Table 6:** Post-varimax rotated component (RC) loadings explained by the PCA model (Adherence Instrument)

Item no.	RC1^a^	RC2 ^a^	RC3 ^a^	RC4 ^a^	RC5 ^a^	h2^b^
1.	-0.100	0.248	0.269	0.078	**0.699**	0.638
2.	0.309	0.141	0.060	-0.027	**0.702**	0.611
3.	**0.771**	-0.062	0.182	0.272	0.009	0.705
4.	**0.840**	0.020	0.157	0.262	-0.032	0.801
5.	**0.782**	-0.022	0.207	0.142	0.025	0.674
6.	**0.770**	-0.078	0.064	0.006	0.128	0.619
7.	**0.819**	0.036	0.030	0.002	0.222	0.722
8.	0.320	0.025	-0.191	**0.723**	0.106	0.674
9.	0.083	0.115	0.196	**0.776**	0.253	0.725
10.	0.309	0.034	**0.709**	0.070	0.196	0.643
11.	0.108	0.067	0.055	0.159	**0.798**	0.682
12.	-0.008	**0.660**	0.020	0.140	0.160	0.482
13.	-0.050	**0.805**	0.060	0.112	0.184	0.701
14.	-0.089	**0.779**	0.223	0.024	0.050	0.667
15.	0.137	0.186	0.358	**0.573**	-0.141	0.529
16.	0.511	0.081	**0.574**	-0.035	0.100	0.608
17.	0.179	0.221	**0.737**	0.113	0.139	0.656
18.	0.265	**0.432**	-0.404	-0.233	-0.042	0.475

Footnote Table 6 shows rotated components (RC) loadings postvarimax rotation via a polychoric matrix; numbers in bold indicate significant loadings for each respective rotated component

^a^ = rotated components 1–5

^b^ = communality (h^2^) of each item explained by the PCA model

Kaiser‒Meyer‒Olkin test: 0.748

Bartlett’s test of sphericity: <0.001

The internal consistency of these components varied, reflecting a range of moderate to strong reliability. Specifically, the reliability coefficients for each component were as follows: RC1 exhibited strong reliability at α = 0.89, RC3 at α = 0.76, and RC2 improved to α = 0.72 after the removal of item 18, which demonstrated low loadings (0.432) and poor communality (0.475), indicating that it was not well-aligned with the intended component (Table 6). This adjustment marked a significant increase from RC2’s initial α = 0.61. Moreover, RC4 and RC5 demonstrated moderate reliability with α = 0.63 and α = 0.69, respectively. The overall scale reliability remained strong at α = 0.84.

## Discussion

In this study, we developed and psychometrically evaluated the Adherence Instrument, which is designed to assess ambulance nurses’ adherence and attitudes towards acute chest pain guidelines, as well as adapting and testing the Attitudes Regarding Practice Guidelines [12] instrument for assessing general attitudes towards guidelines. The findings confirm that both instruments possess robust psychometric properties and good reliability. Notably, the Adherence Instrument demonstrated strong construct validity, and the observed convergent validity between the instruments underscores their efficacy in accurately measuring adherence behaviours and attitudes essential for guideline implementation.

This study’s significant contribution is evident in the development of the Adherence Instrument, which is tailored specifically for ambulance nurses working with acute chest pain guidelines and their involvement during its development and evaluation. This approach is practical and potentially bolsters accurate and reliable adherence measurements [40]. The refinement of the Adherence Instrument from 49 to 18 items is commendable, reinforcing its foundation by retaining only the most pertinent and reliable items [22, 40]. Although this study relied on a sample of 22 ambulance nurses, it revealed a positive correlation between the Adherence Instrument and Self-Reported Adherence measure and the Attitudes Regarding Practice Guidelines instrument, suggesting effective adherence and attitude measurement.

Through cognitive interviewing techniques, we enhanced the face and linguistic validity of both the Adherence Instrument, Self-Reported Adherence measure, and the Attitudes Regarding Practice Guidelines instrument. This process, involving iterative interviews, improved our understanding of the ambulance nurses’ perspectives [18, 19] and helped refine the overall clarity and relevance of the items [41]. Despite challenges in decision-making, items deemed complicated were retained for expert panel review, aiding further refinement. This was particularly apparent in the Attitudes Regarding Practice Guidelines instrument, where no items were eliminated throughout this procedure. During the content validity assessment, essential items in the Self-Reported Adherence measure were retained despite low I-CVI values, ensuring measurement necessity. The S-CVI for this section fell short of the set criterion, potentially influenced by disparities in the expertise and comprehension of the expert panel or the impact of recent shifts in oxygen therapy guidelines [42] on the I-CVI of item number 3. Finally, strong content validity was indicated by acceptable S-CVI values for the Adherence Instrument and the Attitudes Regarding Practice Guidelines, even though one item evaluated below the I-CVI criterion was deemed necessary for the Adherence Instrument measurement [20, 22].

The test-retest results demonstrated strong intrarater reliability, with ICC values exceeding 0.75, indicative of high agreement between repeated measurements [31]. Furthermore, acceptable internal consistency was observed, with Cronbach’s alpha values exceeding 0.70, thus indicating that the items within each instrument are highly correlated and consistently measure the same construct [34]. Despite the Self-Reported Adherence measure exhibiting moderate interrater reliability and a lower Cronbach’s alpha value, potentially due to scale constraints, varied item interrelatedness, or heterogeneous constructs [34], its alpha values was considered acceptable. As the Self-Reported Adherence measurements are based on guidelines [43], further analysis of construct validity may reveal additional constructs that could enhance the scale’s internal consistency [44]. Interrater reliability can be influenced by several factors such as rater bias, item complexity, and the number of response options on an ordinal scale [45]. Hence, despite overall acceptable reliability, item-level test-retest agreement variations highlighted opportunities for instrument reliability improvement and continuous refinement.

The PCA of the Adherence Instrument identified five components: professional evidence-based practice, assessment of symptoms, confidence in skills, clinical autonomy, and guidelines clarity and education. These components account for a substantial part of the variance (64.5%) with an overall strong reliability ordinal α = 0.84, confirming its structural foundation and internal consistency. However, two subscales, RC4 and RC5, showed lower reliabilities (α = 0.63 and α = 0.69, respectively), below the accepted threshold of 0.7, suggesting potential areas for improvement. A notable strength of this PCA is its use of the polychoric correlation matrix, which is ideal for analysing ordinal data, enhancing the robustness of the findings [26]. Furthermore, the implementation of these PCA results could significantly influence the development of targeted posteducational programs and the continuing professional development of ambulance nurses [46, 47], particularly concerning adherence to acute chest pain guidelines. Initiatives might include peer reviews that leverage patient outcomes and professional feedback [46, 48], as well as the development of standardized assessment tools such as the Adherence Instrument. Despite promising preliminary outcomes, the use of checklists in emergency medical services to standardize practices and boost reliability in emergency settings remains underutilized [49]. Evidence suggests that checklists can significantly enhance adherence and improve patient outcomes [49, 50]. The effective dissemination of clear guidelines, coupled with regular updates [6, 8], is crucial to improving adherence rates and enhancing patient outcomes [48].

The findings of this study shed light on the validity and reliability of the Adherence Instrument in measuring both adherence to and attitudes towards guidelines for patients with acute chest pain. The positive correlation between the Adherence Instrument and the Self-Reported Adherence measure suggests effectively measure adherence to guidelines. Additionally, the positive correlation between the Adherence Instrument and the Attitudes Regarding Practice Guidelines instrument indicates that the Adherence instrument also measures attitudes towards these guidelines. The lack of a significant correlation between the Self-Reported Adherence measure and Attitudes Regarding Practice Guidelines instrument indicates that one’s general attitude towards guidelines alone may not impact adherence to acute chest pain guidelines in the prehospital emergency care setting.

While these findings enhance our understanding of the Swedish ambulance service, its international applicability might be constrained by structural and occupational variances. Therefore, future research is essential to evaluate effectiveness of these instruments across different global ambulance service structures to confirm their utility in diverse prehospital care environments.

Furthermore, considering the broader implications of our study, the relationship between Self-Reported Adherence measure and actual clinical outcomes remains an area for further investigation. This exploration is crucial for understanding how well self-reported measures predict real-world behaviours and patient outcomes, which could significantly impact guideline implementation strategies and patient care.

## Conclusion

This study highlights the development and psychometric evaluation of the Adherence Instrument, which measures ambulance nurses’ adherence to and attitudes towards acute chest pain guidelines. The current evaluations suggest that the instrument has promising validity and reliability, though further testing is recommended to confirm these findings. Future research should use this instrument to map adherence and attitudes among ambulance nurses.

## Data Availability

The datasets used and/or analysed during this study can be obtained from the corresponding author upon reasonable request.

## Abbreviations

CVI: Content validity index
ICC: Intraclass correlation coefficient
I-CVI: Item content validity index
KMO: Kaiser–Meyer–Olkin
K_w_: Cohen’s linear weighted kappa
PCA: Principal component analysis
RC: Rotated component
S-CVI: Scale content validity index
